# [*N*,*N*-Bis(diphenyl­phosphan­yl)propanamine-κ^2^ *P*,*P*′]dichloridonickel(II)

**DOI:** 10.1107/S1600536811042760

**Published:** 2011-10-22

**Authors:** Bang-Shao Yin, Tian-Bao Li, Ming-Sheng Yang

**Affiliations:** a College of Chemistry and Chemical Engineering, Hunan Normal University, Changsha, Hunan 410081, People’s Republic of China

## Abstract

In the title complex, [NiCl_2_(C_27_H_27_NP_2_)], the Ni^2+^ ion is coordinated by two chloride ions and two P atoms of the bidentate *N*,*N*-bis­(diphenyl­phosphan­yl)propyl ligand to generate a strongly distorted *cis*-NiCl_2_P_2_ square-planar geometry for the metal ion. A NiP_2_N rhombus occurs within the chelating ligand.

## Related literature

For details of the synthesis, see: Sun *et al.* (2006 ▶). For a related structure, see: Yin *et al.* (2011 ▶).
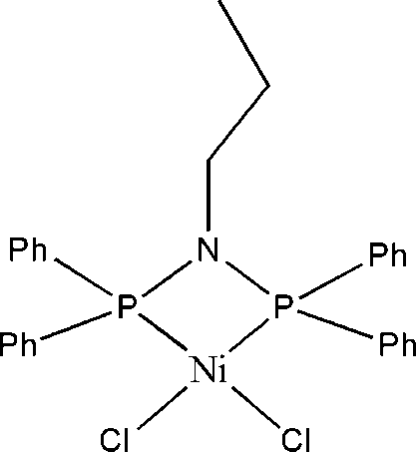


## Experimental

### 

#### Crystal data

[NiCl_2_(C_27_H_27_NP_2_)]
*M* *_r_* = 557.05
Monoclinic, 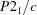

*a* = 10.210 (4) Å
*b* = 19.308 (7) Å
*c* = 15.538 (4) Å
β = 122.669 (18)°
*V* = 2578.5 (15) Å^3^
*Z* = 4
Mo *K*α radiation
μ = 1.10 mm^−1^
*T* = 113 K
0.08 × 0.08 × 0.04 mm

#### Data collection

Rigaku Saturn724 CCD diffractometer
Absorption correction: multi-scan (*CrystalClear*; Rigaku/MSC, 2005 ▶) *T*_min_ = 0.917, *T*_max_ = 0.957
21461 measured reflections
4550 independent reflections
3978 reflections with *I* > 2σ(*I*)
*R*_int_ = 0.076

#### Refinement

*R*[*F* ^2^ > 2σ(*F* ^2^)] = 0.068
*wR*(*F* ^2^) = 0.175
*S* = 1.15
4550 reflections
299 parameters
H-atom parameters constrained
Δρ_max_ = 1.66 e Å^−3^
Δρ_min_ = −0.57 e Å^−3^

### 

Data collection: *CrystalClear* (Rigaku/MSC, 2005 ▶); cell refinement: *CrystalClear*; data reduction: *CrystalClear*; program(s) used to solve structure: *SHELXS97* (Sheldrick, 2008 ▶); program(s) used to refine structure: *SHELXL97* (Sheldrick, 2008 ▶); molecular graphics: *SHELXTL* (Sheldrick, 2008 ▶); software used to prepare material for publication: *CrystalStructure* (Rigaku/MSC, 2005 ▶).

## Supplementary Material

Crystal structure: contains datablock(s) global, I. DOI: 10.1107/S1600536811042760/hb6453sup1.cif

Structure factors: contains datablock(s) I. DOI: 10.1107/S1600536811042760/hb6453Isup2.hkl

Additional supplementary materials:  crystallographic information; 3D view; checkCIF report

## Figures and Tables

**Table:** 

Ni1—P2	2.1244 (16)
Ni1—P1	2.1274 (16)
Ni1—Cl2	2.1964 (17)
Ni1—Cl1	2.1977 (16)

**Table:** 

P2—Ni1—P1	73.41 (5)
P2—Ni1—Cl2	96.11 (5)
P1—Ni1—Cl2	168.01 (6)
P2—Ni1—Cl1	163.08 (6)
P1—Ni1—Cl1	91.96 (6)
Cl2—Ni1—Cl1	99.22 (6)

**Fig. 1.**
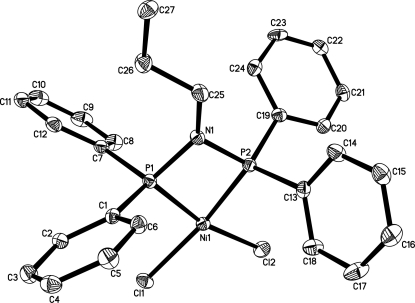
The molecular structure of (I). Displacement ellipsoids are drawn at the 30% probability level and H atoms are shown as small spheres of arbitrary radius.

**Fig. 2.**
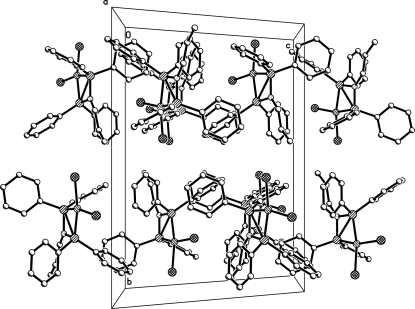
The packing for (I).

## supplementary crystallographic information

### Experimental

The title complex, (I), was prepared according to the literature procedures (Sun *et al.*, 2006).  Red prisms of (I) were grown from slow evaporation of a dichloromethane and hexane solution at room temperature.

### Refinement

All the H atoms were positioned geometrically (C—H = 0.93–0.97 Å) and refined as riding with *U*_iso_(H) = 1.2*U*_eq_(C) or 1.5*U*_eq_(methyl C).

### Crystal data

**Table:**

[NiCl_2_(C_27_H_27_NP_2_)]	*F*(000) = 1152
*M**_r_* = 557.05	*D*_x_ = 1.435 Mg m^−^^3^
Monoclinic, *P*2_1_/*c*	Mo *K*α radiation, λ = 0.71073 Å
Hall symbol: -P 2ybc	Cell parameters from 7237 reflections
*a* = 10.210 (4) Å	θ = 1.9–26.1°
*b* = 19.308 (7) Å	µ = 1.10 mm^−^^1^
*c* = 15.538 (4) Å	*T* = 113 K
β = 122.669 (18)°	Prism, red
*V* = 2578.5 (15) Å^3^	0.08 × 0.08 × 0.04 mm
*Z* = 4	

### Data collection

**Table:**

Rigaku Saturn724 CCD diffractometer	4550 independent reflections
Radiation source: rotating anode	3978 reflections with *I* > 2σ(*I*)
multilayer	*R*_int_ = 0.076
Detector resolution: 14.22 pixels mm^-1^	θ_max_ = 25.0°, θ_min_ = 1.9°
ω and φ scans	*h* = −12→12
Absorption correction: multi-scan (*CrystalClear*; Rigaku/MSC, 2005)	*k* = −22→22
*T*_min_ = 0.917, *T*_max_ = 0.957	*l* = −17→18
21461 measured reflections	

### Refinement

**Table:**

Refinement on *F*^2^	Primary atom site location: structure-invariant direct methods
Least-squares matrix: full	Secondary atom site location: difference Fourier map
*R*[*F*^2^ > 2σ(*F*^2^)] = 0.068	Hydrogen site location: inferred from neighbouring sites
*wR*(*F*^2^) = 0.175	H-atom parameters constrained
*S* = 1.15	*w* = 1/[σ^2^(*F*_o_^2^) + (0.0758*P*)^2^ + 3.0298*P*] where *P* = (*F*_o_^2^ + 2*F*_c_^2^)/3
4550 reflections	(Δ/σ)_max_ < 0.001
299 parameters	Δρ_max_ = 1.66 e Å^−^^3^
0 restraints	Δρ_min_ = −0.57 e Å^−^^3^

### Special details

**Table:**

Geometry. All e.s.d.'s (except the e.s.d. in the dihedral angle between two l.s. planes) are estimated using the full covariance matrix. The cell e.s.d.'s are taken into account individually in the estimation of e.s.d.'s in distances, angles and torsion angles; correlations between e.s.d.'s in cell parameters are only used when they are defined by crystal symmetry. An approximate (isotropic) treatment of cell e.s.d.'s is used for estimating e.s.d.'s involving l.s. planes.
Refinement. Refinement of *F*^2^ against ALL reflections. The weighted *R*-factor *wR* and goodness of fit *S* are based on *F*^2^, conventional *R*-factors *R* are based on *F*, with *F* set to zero for negative *F*^2^. The threshold expression of *F*^2^ > σ(*F*^2^) is used only for calculating *R*-factors(gt) *etc*. and is not relevant to the choice of reflections for refinement. *R*-factors based on *F*^2^ are statistically about twice as large as those based on *F*, and *R*- factors based on ALL data will be even larger.

### Fractional atomic coordinates and isotropic or equivalent isotropic displacement parameters (Å^2^)

**Table:**

	*x*	*y*	*z*	*U*_iso_*/*U*_eq_	
Ni1	0.68919 (7)	0.31831 (3)	0.21970 (5)	0.0212 (2)	
Cl1	0.44166 (14)	0.29800 (7)	0.10697 (10)	0.0306 (3)	
Cl2	0.67979 (15)	0.43135 (7)	0.23126 (10)	0.0330 (3)	
P1	0.74240 (14)	0.21070 (7)	0.23889 (10)	0.0211 (3)	
P2	0.93563 (14)	0.31029 (7)	0.31062 (10)	0.0209 (3)	
N1	0.9378 (4)	0.2224 (2)	0.3110 (3)	0.0218 (9)	
C1	0.6902 (5)	0.1587 (3)	0.1285 (4)	0.0214 (10)	
C2	0.5479 (6)	0.1242 (3)	0.0752 (4)	0.0268 (12)	
H2	0.4800	0.1259	0.0993	0.032*	
C3	0.5054 (6)	0.0877 (3)	−0.0125 (4)	0.0307 (12)	
H3	0.4084	0.0642	−0.0485	0.037*	
C4	0.6052 (6)	0.0851 (3)	−0.0485 (4)	0.0315 (13)	
H4	0.5766	0.0594	−0.1083	0.038*	
C5	0.7447 (6)	0.1200 (3)	0.0033 (4)	0.0327 (13)	
H5	0.8123	0.1184	−0.0211	0.039*	
C6	0.7874 (6)	0.1573 (3)	0.0906 (4)	0.0262 (11)	
H6	0.8829	0.1821	0.1250	0.031*	
C7	0.6744 (5)	0.1657 (3)	0.3101 (4)	0.0248 (11)	
C8	0.6334 (6)	0.2064 (3)	0.3666 (4)	0.0333 (13)	
H8	0.6415	0.2554	0.3666	0.040*	
C9	0.5809 (7)	0.1747 (4)	0.4225 (4)	0.0415 (16)	
H9	0.5494	0.2024	0.4588	0.050*	
C10	0.5737 (6)	0.1036 (4)	0.4260 (5)	0.0435 (16)	
H10	0.5413	0.0825	0.4667	0.052*	
C11	0.6136 (6)	0.0627 (3)	0.3701 (4)	0.0391 (15)	
H11	0.6056	0.0137	0.3711	0.047*	
C12	0.6652 (6)	0.0932 (3)	0.3127 (4)	0.0295 (12)	
H12	0.6941	0.0652	0.2753	0.035*	
C13	1.0345 (6)	0.3415 (3)	0.2503 (4)	0.0247 (11)	
C14	1.1920 (6)	0.3300 (3)	0.2929 (4)	0.0317 (13)	
H14	1.2522	0.3079	0.3576	0.038*	
C15	1.2620 (6)	0.3506 (3)	0.2417 (4)	0.0343 (13)	
H15	1.3699	0.3424	0.2713	0.041*	
C16	1.1762 (7)	0.3828 (3)	0.1482 (4)	0.0364 (14)	
H16	1.2242	0.3960	0.1126	0.044*	
C17	1.0194 (7)	0.3960 (3)	0.1059 (5)	0.0410 (15)	
H17	0.9608	0.4197	0.0425	0.049*	
C18	0.9484 (6)	0.3747 (3)	0.1559 (4)	0.0321 (13)	
H18	0.8404	0.3828	0.1258	0.039*	
C19	1.0500 (5)	0.3412 (3)	0.4406 (4)	0.0239 (11)	
C20	1.0872 (6)	0.4110 (3)	0.4589 (4)	0.0265 (11)	
H20	1.0581	0.4413	0.4031	0.032*	
C21	1.1660 (6)	0.4372 (3)	0.5572 (4)	0.0313 (12)	
H21	1.1881	0.4853	0.5686	0.038*	
C22	1.2134 (6)	0.3926 (3)	0.6401 (4)	0.0291 (12)	
H22	1.2697	0.4101	0.7079	0.035*	
C23	1.1778 (6)	0.3232 (3)	0.6224 (4)	0.0334 (13)	
H23	1.2114	0.2930	0.6789	0.040*	
C24	1.0937 (6)	0.2961 (3)	0.5239 (4)	0.0283 (12)	
H24	1.0662	0.2485	0.5128	0.034*	
C25	1.0738 (6)	0.1766 (3)	0.3458 (4)	0.0299 (12)	
H25A	1.1635	0.1957	0.4097	0.036*	
H25B	1.1010	0.1767	0.2936	0.036*	
C26	1.0480 (6)	0.1034 (3)	0.3647 (5)	0.0347 (13)	
H26A	0.9621	0.0829	0.3003	0.042*	
H26B	1.0172	0.1027	0.4152	0.042*	
C27	1.1960 (6)	0.0599 (3)	0.4050 (4)	0.0322 (13)	
H27A	1.2205	0.0566	0.3522	0.048*	
H27B	1.1791	0.0133	0.4224	0.048*	
H27C	1.2828	0.0819	0.4661	0.048*	

### Atomic displacement parameters (Å^2^)

**Table:**

	*U*^11^	*U*^22^	*U*^33^	*U*^12^	*U*^13^	*U*^23^
Ni1	0.0193 (4)	0.0231 (4)	0.0222 (4)	0.0025 (3)	0.0119 (3)	0.0018 (3)
Cl1	0.0179 (6)	0.0413 (8)	0.0293 (7)	0.0024 (5)	0.0106 (5)	0.0007 (6)
Cl2	0.0362 (8)	0.0258 (7)	0.0377 (8)	0.0063 (5)	0.0205 (6)	0.0023 (6)
P1	0.0193 (7)	0.0236 (7)	0.0216 (7)	−0.0009 (5)	0.0118 (5)	−0.0001 (5)
P2	0.0194 (7)	0.0213 (7)	0.0219 (7)	0.0008 (5)	0.0112 (5)	0.0004 (5)
N1	0.018 (2)	0.023 (2)	0.024 (2)	0.0008 (17)	0.0112 (18)	−0.0022 (18)
C1	0.022 (3)	0.017 (3)	0.026 (3)	0.003 (2)	0.013 (2)	0.001 (2)
C2	0.025 (3)	0.029 (3)	0.023 (3)	−0.002 (2)	0.012 (2)	0.001 (2)
C3	0.028 (3)	0.033 (3)	0.030 (3)	−0.003 (2)	0.014 (2)	−0.002 (2)
C4	0.034 (3)	0.033 (3)	0.025 (3)	0.005 (2)	0.014 (2)	−0.001 (2)
C5	0.034 (3)	0.039 (3)	0.030 (3)	−0.002 (2)	0.021 (3)	−0.004 (3)
C6	0.027 (3)	0.027 (3)	0.027 (3)	−0.003 (2)	0.017 (2)	−0.001 (2)
C7	0.020 (3)	0.033 (3)	0.020 (3)	−0.001 (2)	0.010 (2)	0.004 (2)
C8	0.033 (3)	0.041 (3)	0.031 (3)	0.003 (3)	0.021 (3)	0.001 (3)
C9	0.034 (3)	0.071 (5)	0.027 (3)	0.008 (3)	0.021 (3)	0.008 (3)
C10	0.026 (3)	0.073 (5)	0.034 (3)	−0.001 (3)	0.017 (3)	0.017 (3)
C11	0.026 (3)	0.047 (4)	0.034 (3)	−0.009 (3)	0.009 (3)	0.012 (3)
C12	0.019 (3)	0.038 (3)	0.028 (3)	−0.002 (2)	0.010 (2)	0.003 (2)
C13	0.028 (3)	0.023 (3)	0.026 (3)	−0.005 (2)	0.016 (2)	−0.003 (2)
C14	0.025 (3)	0.043 (4)	0.025 (3)	−0.002 (2)	0.013 (2)	−0.003 (2)
C15	0.028 (3)	0.048 (4)	0.033 (3)	−0.005 (3)	0.021 (3)	−0.006 (3)
C16	0.044 (3)	0.044 (4)	0.038 (3)	0.003 (3)	0.033 (3)	0.002 (3)
C17	0.049 (4)	0.047 (4)	0.038 (3)	0.020 (3)	0.031 (3)	0.018 (3)
C18	0.030 (3)	0.041 (4)	0.028 (3)	0.009 (2)	0.017 (2)	0.006 (2)
C19	0.020 (3)	0.028 (3)	0.025 (3)	0.004 (2)	0.013 (2)	0.002 (2)
C20	0.029 (3)	0.027 (3)	0.027 (3)	0.000 (2)	0.017 (2)	0.002 (2)
C21	0.029 (3)	0.032 (3)	0.031 (3)	−0.008 (2)	0.016 (2)	−0.010 (2)
C22	0.025 (3)	0.040 (3)	0.024 (3)	0.000 (2)	0.014 (2)	−0.005 (2)
C23	0.036 (3)	0.047 (4)	0.018 (3)	0.012 (3)	0.015 (2)	0.012 (2)
C24	0.029 (3)	0.026 (3)	0.037 (3)	0.001 (2)	0.022 (3)	0.001 (2)
C25	0.020 (3)	0.025 (3)	0.039 (3)	0.003 (2)	0.013 (2)	−0.002 (2)
C26	0.030 (3)	0.024 (3)	0.045 (3)	0.000 (2)	0.017 (3)	−0.002 (3)
C27	0.034 (3)	0.028 (3)	0.040 (3)	0.008 (2)	0.023 (3)	0.006 (3)

### Geometric parameters (Å, °)

**Table:**

Ni1—P2	2.1244 (16)	C12—H12	0.9500
Ni1—P1	2.1274 (16)	C13—C14	1.387 (7)
Ni1—Cl2	2.1964 (17)	C13—C18	1.395 (7)
Ni1—Cl1	2.1977 (16)	C14—C15	1.385 (7)
P1—N1	1.695 (4)	C14—H14	0.9500
P1—C1	1.802 (5)	C15—C16	1.376 (8)
P1—C7	1.815 (5)	C15—H15	0.9500
P1—P2	2.5413 (19)	C16—C17	1.387 (8)
P2—N1	1.697 (4)	C16—H16	0.9500
P2—C19	1.803 (5)	C17—C18	1.380 (8)
P2—C13	1.811 (5)	C17—H17	0.9500
N1—C25	1.480 (6)	C18—H18	0.9500
C1—C2	1.393 (7)	C19—C20	1.389 (7)
C1—C6	1.401 (7)	C19—C24	1.418 (7)
C2—C3	1.380 (7)	C20—C21	1.381 (7)
C2—H2	0.9500	C20—H20	0.9500
C3—C4	1.403 (7)	C21—C22	1.400 (8)
C3—H3	0.9500	C21—H21	0.9500
C4—C5	1.376 (8)	C22—C23	1.377 (8)
C4—H4	0.9500	C22—H22	0.9500
C5—C6	1.382 (7)	C23—C24	1.390 (8)
C5—H5	0.9500	C23—H23	0.9500
C6—H6	0.9500	C24—H24	0.9500
C7—C8	1.401 (7)	C25—C26	1.495 (7)
C7—C12	1.404 (8)	C25—H25A	0.9900
C8—C9	1.385 (8)	C25—H25B	0.9900
C8—H8	0.9500	C26—C27	1.536 (7)
C9—C10	1.379 (9)	C26—H26A	0.9900
C9—H9	0.9500	C26—H26B	0.9900
C10—C11	1.387 (9)	C27—H27A	0.9800
C10—H10	0.9500	C27—H27B	0.9800
C11—C12	1.390 (7)	C27—H27C	0.9800
C11—H11	0.9500		
			
P2—Ni1—P1	73.41 (5)	C7—C12—H12	120.1
P2—Ni1—Cl2	96.11 (5)	C14—C13—C18	119.1 (5)
P1—Ni1—Cl2	168.01 (6)	C14—C13—P2	121.8 (4)
P2—Ni1—Cl1	163.08 (6)	C18—C13—P2	119.1 (4)
P1—Ni1—Cl1	91.96 (6)	C15—C14—C13	120.4 (5)
Cl2—Ni1—Cl1	99.22 (6)	C15—C14—H14	119.8
N1—P1—Ni1	94.77 (15)	C13—C14—H14	119.8
N1—P2—Ni1	94.82 (14)	C16—C15—C14	120.3 (5)
P1—N1—P2	97.0 (2)	C16—C15—H15	119.8
N1—P1—C1	109.9 (2)	C14—C15—H15	119.8
N1—P1—C7	111.2 (2)	C15—C16—C17	119.8 (5)
C1—P1—C7	107.5 (2)	C15—C16—H16	120.1
C1—P1—Ni1	119.23 (17)	C17—C16—H16	120.1
C7—P1—Ni1	113.62 (18)	C18—C17—C16	120.1 (5)
N1—P2—C19	109.1 (2)	C18—C17—H17	119.9
N1—P2—C13	108.9 (2)	C16—C17—H17	119.9
C19—P2—C13	105.3 (2)	C17—C18—C13	120.3 (5)
C19—P2—Ni1	122.63 (16)	C17—C18—H18	119.9
C13—P2—Ni1	115.01 (18)	C13—C18—H18	119.9
C25—N1—P1	134.3 (3)	C20—C19—C24	119.5 (5)
C25—N1—P2	127.4 (3)	C20—C19—P2	119.4 (4)
C2—C1—C6	119.3 (5)	C24—C19—P2	121.0 (4)
C2—C1—P1	120.6 (4)	C21—C20—C19	120.9 (5)
C6—C1—P1	119.9 (4)	C21—C20—H20	119.6
C3—C2—C1	120.1 (5)	C19—C20—H20	119.6
C3—C2—H2	119.9	C20—C21—C22	119.9 (5)
C1—C2—H2	119.9	C20—C21—H21	120.1
C2—C3—C4	120.2 (5)	C22—C21—H21	120.1
C2—C3—H3	119.9	C23—C22—C21	119.5 (5)
C4—C3—H3	119.9	C23—C22—H22	120.3
C5—C4—C3	119.6 (5)	C21—C22—H22	120.3
C5—C4—H4	120.2	C22—C23—C24	121.7 (5)
C3—C4—H4	120.2	C22—C23—H23	119.2
C4—C5—C6	120.5 (5)	C24—C23—H23	119.2
C4—C5—H5	119.7	C23—C24—C19	118.5 (5)
C6—C5—H5	119.7	C23—C24—H24	120.7
C5—C6—C1	120.2 (5)	C19—C24—H24	120.7
C5—C6—H6	119.9	N1—C25—C26	114.0 (4)
C1—C6—H6	119.9	N1—C25—H25A	108.7
C8—C7—C12	119.7 (5)	C26—C25—H25A	108.7
C8—C7—P1	117.1 (4)	N1—C25—H25B	108.7
C12—C7—P1	123.2 (4)	C26—C25—H25B	108.7
C9—C8—C7	119.5 (6)	H25A—C25—H25B	107.6
C9—C8—H8	120.2	C25—C26—C27	111.0 (4)
C7—C8—H8	120.2	C25—C26—H26A	109.4
C10—C9—C8	120.7 (6)	C27—C26—H26A	109.4
C10—C9—H9	119.6	C25—C26—H26B	109.4
C8—C9—H9	119.6	C27—C26—H26B	109.4
C9—C10—C11	120.2 (5)	H26A—C26—H26B	108.0
C9—C10—H10	119.9	C26—C27—H27A	109.5
C11—C10—H10	119.9	C26—C27—H27B	109.5
C10—C11—C12	120.1 (6)	H27A—C27—H27B	109.5
C10—C11—H11	120.0	C26—C27—H27C	109.5
C12—C11—H11	120.0	H27A—C27—H27C	109.5
C11—C12—C7	119.7 (5)	H27B—C27—H27C	109.5
C11—C12—H12	120.1		
			
P2—Ni1—P1—N1	−0.03 (14)	P1—C1—C2—C3	176.5 (4)
Cl2—Ni1—P1—N1	−29.8 (3)	C1—C2—C3—C4	−0.1 (8)
Cl1—Ni1—P1—N1	171.31 (15)	C2—C3—C4—C5	−0.9 (8)
P2—Ni1—P1—C1	−116.06 (19)	C3—C4—C5—C6	0.2 (9)
Cl2—Ni1—P1—C1	−145.8 (3)	C4—C5—C6—C1	1.4 (8)
Cl1—Ni1—P1—C1	55.28 (19)	C2—C1—C6—C5	−2.3 (8)
P2—Ni1—P1—C7	115.61 (19)	P1—C1—C6—C5	−177.2 (4)
Cl2—Ni1—P1—C7	85.9 (3)	N1—P1—C7—C8	89.3 (4)
Cl1—Ni1—P1—C7	−73.05 (18)	C1—P1—C7—C8	−150.3 (4)
Cl2—Ni1—P1—P2	−29.7 (3)	Ni1—P1—C7—C8	−16.2 (5)
Cl1—Ni1—P1—P2	171.35 (6)	P2—P1—C7—C8	44.3 (5)
P1—Ni1—P2—N1	0.03 (14)	N1—P1—C7—C12	−89.2 (4)
Cl2—Ni1—P2—N1	174.09 (14)	C1—P1—C7—C12	31.2 (5)
Cl1—Ni1—P2—N1	−31.1 (3)	Ni1—P1—C7—C12	165.4 (4)
P1—Ni1—P2—C19	−116.3 (2)	P2—P1—C7—C12	−134.2 (4)
Cl2—Ni1—P2—C19	57.7 (2)	C12—C7—C8—C9	−1.4 (8)
Cl1—Ni1—P2—C19	−147.4 (3)	P1—C7—C8—C9	−180.0 (4)
P1—Ni1—P2—C13	113.49 (19)	C7—C8—C9—C10	2.3 (9)
Cl2—Ni1—P2—C13	−72.46 (19)	C8—C9—C10—C11	−2.5 (9)
Cl1—Ni1—P2—C13	82.4 (3)	C9—C10—C11—C12	1.8 (8)
Cl2—Ni1—P2—P1	174.05 (6)	C10—C11—C12—C7	−1.0 (8)
Cl1—Ni1—P2—P1	−31.1 (2)	C8—C7—C12—C11	0.8 (7)
C1—P1—P2—N1	−78.3 (3)	P1—C7—C12—C11	179.2 (4)
C7—P1—P2—N1	84.2 (3)	N1—P2—C13—C14	−64.9 (5)
Ni1—P1—P2—N1	−179.9 (2)	C19—P2—C13—C14	52.0 (5)
N1—P1—P2—C19	−74.5 (3)	Ni1—P2—C13—C14	−169.8 (4)
C1—P1—P2—C19	−152.8 (3)	P1—P2—C13—C14	−108.7 (4)
C7—P1—P2—C19	9.7 (3)	N1—P2—C13—C18	111.7 (4)
Ni1—P1—P2—C19	105.5 (2)	C19—P2—C13—C18	−131.3 (4)
N1—P1—P2—C13	81.5 (3)	Ni1—P2—C13—C18	6.8 (5)
C1—P1—P2—C13	3.2 (3)	P1—P2—C13—C18	67.9 (5)
C7—P1—P2—C13	165.7 (3)	C18—C13—C14—C15	−0.8 (8)
Ni1—P1—P2—C13	−98.5 (2)	P2—C13—C14—C15	175.8 (4)
N1—P1—P2—Ni1	179.9 (2)	C13—C14—C15—C16	0.2 (9)
C1—P1—P2—Ni1	101.7 (2)	C14—C15—C16—C17	1.3 (9)
C7—P1—P2—Ni1	−95.8 (2)	C15—C16—C17—C18	−2.2 (9)
C1—P1—N1—C25	−43.7 (5)	C16—C17—C18—C13	1.7 (9)
C7—P1—N1—C25	75.2 (5)	C14—C13—C18—C17	−0.1 (8)
Ni1—P1—N1—C25	−167.2 (5)	P2—C13—C18—C17	−176.9 (5)
P2—P1—N1—C25	−167.2 (6)	N1—P2—C19—C20	169.1 (4)
C1—P1—N1—P2	123.5 (2)	C13—P2—C19—C20	52.3 (4)
C7—P1—N1—P2	−117.6 (2)	Ni1—P2—C19—C20	−81.8 (4)
Ni1—P1—N1—P2	0.04 (17)	P1—P2—C19—C20	−148.4 (3)
C19—P2—N1—C25	−64.5 (5)	N1—P2—C19—C24	−15.1 (4)
C13—P2—N1—C25	50.0 (5)	C13—P2—C19—C24	−131.9 (4)
Ni1—P2—N1—C25	168.5 (4)	Ni1—P2—C19—C24	94.0 (4)
P1—P2—N1—C25	168.5 (5)	P1—P2—C19—C24	27.4 (5)
C19—P2—N1—P1	127.0 (2)	C24—C19—C20—C21	−0.1 (7)
C13—P2—N1—P1	−118.5 (2)	P2—C19—C20—C21	175.8 (4)
Ni1—P2—N1—P1	−0.04 (17)	C19—C20—C21—C22	1.9 (8)
N1—P1—C1—C2	160.0 (4)	C20—C21—C22—C23	−1.4 (8)
C7—P1—C1—C2	38.9 (5)	C21—C22—C23—C24	−0.9 (8)
Ni1—P1—C1—C2	−92.2 (4)	C22—C23—C24—C19	2.7 (8)
P2—P1—C1—C2	−156.3 (3)	C20—C19—C24—C23	−2.2 (7)
N1—P1—C1—C6	−25.2 (5)	P2—C19—C24—C23	−178.0 (4)
C7—P1—C1—C6	−146.3 (4)	P1—N1—C25—C26	−32.8 (7)
Ni1—P1—C1—C6	82.6 (4)	P2—N1—C25—C26	163.3 (4)
P2—P1—C1—C6	18.5 (5)	N1—C25—C26—C27	−177.5 (4)
C6—C1—C2—C3	1.6 (8)		
